# 1-Acetyl-5,6-dimethoxy­indoline at 123 K

**DOI:** 10.1107/S1600536808017376

**Published:** 2008-06-19

**Authors:** Xiang-Wei Cheng

**Affiliations:** a Zhejiang Police College Experience Center, Zhejiang Police College, Hangzhou 310053, People’s Republic of China

## Abstract

In the title compound, C_12_H_15_NO_3_, all C, N and O atoms lie in a mirror plane. An intramolecular C—H⋯O hydrogen bond is present.

## Related literature

For the synthesis, see: Kuwano *et al.* (2006 ▶). For general background, see: Fernandez *et al.* (2006 ▶); Amit *et al.* (1976 ▶). For a related structure, see: Moreno *et al.* (1998 ▶).
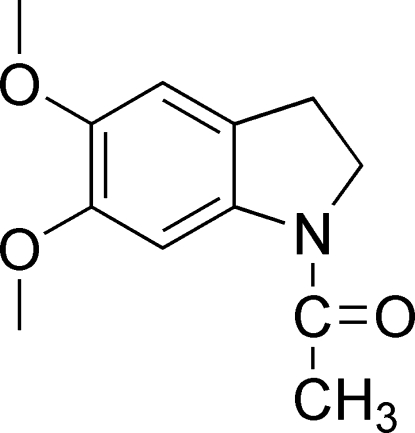


## Experimental

### 

#### Crystal data

C_12_H_15_NO_3_
*M* *_r_* = 221.25
Orthorhombic, 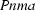

*a* = 18.541 (4) Å
*b* = 6.9572 (15) Å
*c* = 8.5582 (17) Å
*V* = 1103.9 (4) Å^3^
*Z* = 4
Mo *K*α radiation
μ = 0.10 mm^−1^
*T* = 123 (2) K
0.29 × 0.26 × 0.25 mm

#### Data collection

Bruker SMART CCD area-detector diffractometer
Absorption correction: multi-scan (*SADABS*; Bruker, 2002 ▶) *T*_min_ = 0.963, *T*_max_ = 0.976
11013 measured reflections
1054 independent reflections
964 reflections with *I* > 2σ(*I*)
*R*_int_ = 0.028

#### Refinement

*R*[*F* ^2^ > 2σ(*F* ^2^)] = 0.057
*wR*(*F* ^2^) = 0.148
*S* = 1.40
1054 reflections
97 parameters
6 restraints
H-atom parameters constrained
Δρ_max_ = 0.41 e Å^−3^
Δρ_min_ = −0.59 e Å^−3^

### 

Data collection: *SMART* (Bruker, 2002 ▶); cell refinement: *SAINT* (Bruker, 2002 ▶); data reduction: *SAINT*; program(s) used to solve structure: *SHELXS97* (Sheldrick, 2008 ▶); program(s) used to refine structure: *SHELXL97* (Sheldrick, 2008 ▶); molecular graphics: *SHELXTL* (Sheldrick, 2008 ▶); software used to prepare material for publication: *SHELXTL*.

## Supplementary Material

Crystal structure: contains datablocks global, I. DOI: 10.1107/S1600536808017376/wk2084sup1.cif

Structure factors: contains datablocks I. DOI: 10.1107/S1600536808017376/wk2084Isup2.hkl

Additional supplementary materials:  crystallographic information; 3D view; checkCIF report

## Figures and Tables

**Table 1:** Hydrogen-bond geometry (Å, °)

*D*—H⋯*A*	*D*—H	H⋯*A*	*D*⋯*A*	*D*—H⋯*A*
C5—H5⋯O3	0.95	2.30	2.861 (2)	117

**Fig. 1.**
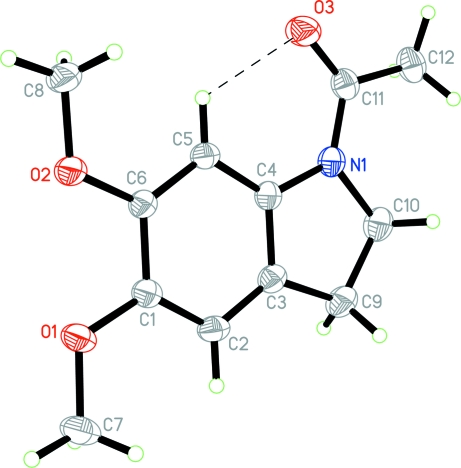
Molecular structure of the title compound, showing 30% probability displacement ellipsoids and the atomic numbering.

## supplementary crystallographic information

### Comment

The indoline cores have attracted particular attention in recent years due to their presence in a great variety of natural products, biologically active alkaloids and pharmaceuticals (Fernandez *et al.*,  2006). Some nitro derivative compounds of 1-acetyl-indoline can undergo photosolvolysis which points to some possible use in the synthesis of peptides (Amit *et al.*,  1976). Here the crystal structure of the title compound is reported.

The title molecule (Fig.1), displays mirror symmetry , with all  C, N atom and O atoms lying in the mirror plane.

### Experimental

The title compound was prepared according to the literature method (Kuwano *et al.*, 2006). Crystals suitable for X-ray analysis were obtained by slow evaporation of an isopropanol solution at 295 K.

### Refinement

H atoms were positioned geometrically (C-H = 0.95-0.99 Å) and refined using a riding model, with *U*_iso_(H) = 1.2–1.5*U*_eq_(C).

### Crystal data

**Table:**

C_12_H_15_NO_3_	*F*_000_ = 472
*M**_r_* = 221.25	*D*_x_ = 1.331 Mg m^−^^3^
Orthorhombic, *P**n**m**a*	Mo *K*α radiation λ = 0.71073 Å
Hall symbol: -P 2ac 2n	Cell parameters from 1054 reflections
*a* = 18.541 (4) Å	θ = 2.2–25.0º
*b* = 6.9572 (15) Å	µ = 0.10 mm^−^^1^
*c* = 8.5582 (17) Å	*T* = 123 (2) K
*V* = 1103.9 (4) Å^3^	Block, colourless
*Z* = 4	0.29 × 0.26 × 0.25 mm

### Data collection

**Table:**

Bruker SMART CCD area-detector diffractometer	1054 independent reflections
Radiation source: fine-focus sealed tube	964 reflections with *I* > 2σ(*I*)
Monochromator: graphite	*R*_int_ = 0.028
*T* = 123(2) K	θ_max_ = 25.0º
ω scans	θ_min_ = 2.2º
Absorption correction: multi-scan(SADABS; Bruker, 2002)	*h* = −20→22
*T*_min_ = 0.963, *T*_max_ = 0.976	*k* = −8→7
11013 measured reflections	*l* = −10→10

### Refinement

**Table:**

Refinement on *F*^2^	Secondary atom site location: difference Fourier map
Least-squares matrix: full	Hydrogen site location: inferred from neighbouring sites
*R*[*F*^2^ > 2σ(*F*^2^)] = 0.057	H-atom parameters constrained
*wR*(*F*^2^) = 0.148	*w* = 1/[σ^2^(*F*_o_^2^) + (0.0677*P*)^2^ + 0.2*P*] where *P* = (*F*_o_^2^ + 2*F*_c_^2^)/3
*S* = 1.40	(Δ/σ)_max_ < 0.001
1054 reflections	Δρ_max_ = 0.41 e Å^−^^3^
97 parameters	Δρ_min_ = −0.59 e Å^−^^3^
6 restraints	Extinction correction: none
Primary atom site location: structure-invariant direct methods	

### Special details

**Table:**

Geometry. All esds (except the esd in the dihedral angle between two l.s. planes) are estimated using the full covariance matrix. The cell esds are taken into account individually in the estimation of esds in distances, angles and torsion angles; correlations between esds in cell parameters are only used when they are defined by crystal symmetry. An approximate (isotropic) treatment of cell esds is used for estimating esds involving l.s. planes.
Refinement. Refinement of F^2^ against ALL reflections. The weighted R-factor wR and goodness of fit S are based on F^2^, conventional R-factors R are based on F, with F set to zero for negative F^2^. The threshold expression of F^2^ > 2sigma(F^2^) is used only for calculating R-factors(gt) etc. and is not relevant to the choice of reflections for refinement. R-factors based on F^2^ are statistically about twice as large as those based on F, and R- factors based on ALL data will be even larger.

### Fractional atomic coordinates and isotropic or equivalent isotropic displacement parameters (Å^2^)

**Table:**

	*x*	*y*	*z*	*U*_iso_*/*U*_eq_	Occ. (<1)
O2	0.74869 (9)	0.2500	0.6594 (2)	0.0501 (6)	
O1	0.78145 (11)	0.2500	0.9506 (2)	0.0526 (6)	
O3	0.95611 (11)	0.2500	0.2934 (2)	0.0602 (7)	
N1	1.00964 (12)	0.2500	0.5307 (3)	0.0412 (6)	
C3	0.96484 (14)	0.2500	0.7812 (3)	0.0399 (7)	
C4	0.94640 (14)	0.2500	0.6247 (3)	0.0366 (6)	
C6	0.82118 (14)	0.2500	0.6909 (3)	0.0369 (6)	
C11	1.01159 (15)	0.2500	0.3721 (3)	0.0439 (7)	
C5	0.87457 (14)	0.2500	0.5772 (3)	0.0375 (6)	
H5	0.8626	0.2500	0.4717	0.045*	
C1	0.83923 (14)	0.2500	0.8506 (3)	0.0397 (7)	
C2	0.91147 (14)	0.2500	0.8952 (3)	0.0424 (7)	
H2	0.9239	0.2500	1.0005	0.051*	
C12	1.08471 (16)	0.2500	0.2962 (4)	0.0529 (8)	
H12A	1.1214	0.2500	0.3754	0.079*	
H12B	1.0897	0.3627	0.2324	0.079*	0.50
H12C	1.0897	0.1373	0.2324	0.079*	0.50
C9	1.04564 (15)	0.2500	0.8003 (4)	0.0515 (8)	
H9A	1.0616	0.1365	0.8563	0.062*	0.50
H9B	1.0616	0.3635	0.8563	0.062*	0.50
C8	0.72852 (17)	0.2500	0.4986 (3)	0.0595 (9)	
H8A	0.6769	0.2500	0.4902	0.089*	
H8B	0.7475	0.1373	0.4487	0.089*	0.50
H8C	0.7475	0.3627	0.4487	0.089*	0.50
C10	1.07441 (15)	0.2500	0.6328 (4)	0.0533 (8)	
H10A	1.1036	0.3633	0.6137	0.064*	0.50
H10B	1.1036	0.1367	0.6137	0.064*	0.50
C7	0.79744 (19)	0.2500	1.1138 (3)	0.0658 (10)	
H7A	0.7533	0.2500	1.1723	0.099*	
H7B	0.8249	0.3627	1.1394	0.099*	0.50
H7C	0.8249	0.1373	1.1394	0.099*	0.50

### Atomic displacement parameters (Å^2^)

**Table:**

	*U*^11^	*U*^22^	*U*^33^	*U*^12^	*U*^13^	*U*^23^
O2	0.0326 (11)	0.0844 (15)	0.0334 (10)	0.000	−0.0007 (7)	0.000
O1	0.0446 (12)	0.0827 (15)	0.0304 (10)	0.000	0.0041 (8)	0.000
O3	0.0465 (13)	0.0931 (18)	0.0410 (12)	0.000	0.0042 (9)	0.000
N1	0.0325 (12)	0.0470 (14)	0.0440 (13)	0.000	0.0020 (9)	0.000
C3	0.0377 (15)	0.0415 (14)	0.0406 (15)	0.000	−0.0061 (11)	0.000
C4	0.0342 (14)	0.0362 (13)	0.0395 (14)	0.000	0.0002 (11)	0.000
C6	0.0317 (13)	0.0443 (14)	0.0348 (13)	0.000	−0.0014 (10)	0.000
C11	0.0416 (16)	0.0437 (15)	0.0463 (14)	0.000	0.0065 (13)	0.000
C5	0.0372 (14)	0.0436 (14)	0.0317 (12)	0.000	−0.0019 (11)	0.000
C1	0.0397 (15)	0.0449 (15)	0.0345 (13)	0.000	0.0013 (11)	0.000
C2	0.0474 (16)	0.0484 (16)	0.0313 (13)	0.000	−0.0074 (11)	0.000
C12	0.0473 (18)	0.0518 (17)	0.0595 (18)	0.000	0.0154 (14)	0.000
C9	0.0392 (17)	0.0622 (19)	0.0532 (18)	0.000	−0.0133 (13)	0.000
C8	0.0360 (16)	0.105 (3)	0.0374 (15)	0.000	−0.0043 (12)	0.000
C10	0.0319 (15)	0.0640 (19)	0.0639 (19)	0.000	−0.0025 (13)	0.000
C7	0.061 (2)	0.105 (3)	0.0311 (14)	0.000	0.0030 (14)	0.000

### Geometric parameters (Å, °)

**Table:**

O2—C6	1.371 (3)	C1—C2	1.393 (4)
O2—C8	1.426 (3)	C2—H2	0.9300
O1—C1	1.371 (3)	C12—H12A	0.9600
O1—C7	1.428 (3)	C12—H12B	0.9600
O3—C11	1.230 (3)	C12—H12C	0.9600
N1—C11	1.358 (4)	C9—C10	1.530 (5)
N1—C4	1.422 (3)	C9—H9A	0.9700
N1—C10	1.485 (4)	C9—H9B	0.9700
C3—C4	1.383 (4)	C8—H8A	0.9600
C3—C2	1.389 (4)	C8—H8B	0.9600
C3—C9	1.507 (4)	C8—H8C	0.9600
C4—C5	1.392 (4)	C10—H10A	0.9700
C6—C5	1.388 (4)	C10—H10B	0.9700
C6—C1	1.408 (4)	C7—H7A	0.9600
C11—C12	1.503 (4)	C7—H7B	0.9600
C5—H5	0.9300	C7—H7C	0.9600
			
C6—O2—C8	116.6 (2)	H12A—C12—H12B	109.5
C1—O1—C7	116.6 (2)	C11—C12—H12C	109.5
C11—N1—C4	126.0 (2)	H12A—C12—H12C	109.5
C11—N1—C10	124.5 (2)	H12B—C12—H12C	109.5
C4—N1—C10	109.5 (2)	C3—C9—C10	104.2 (2)
C4—C3—C2	120.3 (2)	C3—C9—H9A	110.9
C4—C3—C9	110.5 (2)	C10—C9—H9A	110.9
C2—C3—C9	129.2 (2)	C3—C9—H9B	110.9
C3—C4—C5	121.3 (2)	C10—C9—H9B	110.9
C3—C4—N1	110.1 (2)	H9A—C9—H9B	108.9
C5—C4—N1	128.6 (2)	O2—C8—H8A	109.5
O2—C6—C5	124.2 (2)	O2—C8—H8B	109.5
O2—C6—C1	115.1 (2)	H8A—C8—H8B	109.5
C5—C6—C1	120.7 (2)	O2—C8—H8C	109.5
O3—C11—N1	121.7 (2)	H8A—C8—H8C	109.5
O3—C11—C12	121.2 (3)	H8B—C8—H8C	109.5
N1—C11—C12	117.1 (3)	N1—C10—C9	105.6 (2)
C6—C5—C4	118.5 (2)	N1—C10—H10A	110.6
C6—C5—H5	120.7	C9—C10—H10A	110.6
C4—C5—H5	120.7	N1—C10—H10B	110.6
O1—C1—C2	125.5 (2)	C9—C10—H10B	110.6
O1—C1—C6	114.8 (2)	H10A—C10—H10B	108.7
C2—C1—C6	119.6 (2)	O1—C7—H7A	109.5
C3—C2—C1	119.5 (2)	O1—C7—H7B	109.5
C3—C2—H2	120.2	H7A—C7—H7B	109.5
C1—C2—H2	120.2	O1—C7—H7C	109.5
C11—C12—H12A	109.5	H7A—C7—H7C	109.5
C11—C12—H12B	109.5	H7B—C7—H7C	109.5

### Hydrogen-bond geometry (Å, °)

**Table:**

*D*—H···*A*	*D*—H	H···*A*	*D*···*A*	*D*—H···*A*
C5—H5···O3	0.95	2.30	2.861 (2)	117
